# Prevalence of gastroesophageal reflux disease: a population-based cross-sectional study in southern Chile

**DOI:** 10.1093/gastro/goaa002

**Published:** 2020-02-19

**Authors:** Carlos Manterola, Luis Grande, Luis Bustos, Tamara Otzen

**Affiliations:** g1 Center of Morphological and Surgical Studies, Universidad de La Frontera, Temuco, Chile; g2 Department of Surgery, Universidad de La Frontera, Temuco, Chile; g3 Department of Surgery, Hospital del Mar, Universitat Autònoma de Barcelona, Barcelona, Spain; g4 Department of Public Health, Universidad de La Frontera, Temuco, Chile

**Keywords:** gastroesophageal reflux disease, prevalence, cross-sectional studies, evidence-based medicine, clinical epidemiology

## Abstract

**Background:**

This study assessed the prevalence of gastroesophageal reflux disease (GERD) in a general adult population from Temuco in southern Chile. The association of GERD with demographic variables was also examined.

**Methods:**

A cross-sectional study among the general population of Temuco in southern Chile was conducted in 2017, using a validated and reliable questionnaire for detecting GERD. The urban area of Temuco, with a population of 245,317 inhabitants (2002 census), was divided into four zones, which were representative of the socioeconomic sectors of the city. The sample size was estimated assuming a prevalence of 52.8%, an accuracy of 3.0%, a confidence level of 95.0%, and a design effect of 1.15. Area sampling was used to build clusters. The prevalence of GERD was determined and associated factors were studied by means of bivariate and multivariate analyses.

**Results:**

A total of 1,069 subjects (47.9% women, median age 40 years) from the selected subareas were interviewed. The prevalence of GERD was 44.8%. The most frequently reported symptom was regurgitation (54.8%). One-third of subjects took medication to control symptoms and was considered ‘sick’ by the instrument, although >68% of them had never sought medical consultation. There was a significant association between GERD and age (*P *<* *0.001) and female gender (*P *=* *0.001).

**Conclusions:**

In this population-based study, the prevalence of GERD was high (44.8%). GERD was associated with age and female gender.

## Introduction

Gastroesophageal reflux disease (GERD) is a frequent cause of consultation. Typical symptoms, such as heartburn and regurgitation are common in the community, occurring in up to 33% of the adult population. It has been estimated that 30% of symptomatic subjects have esophagitis and that 70% require regular treatment for the control of symptoms, which can strongly affect their quality of life [1]. There are a few reports on the prevalence of GERD in the general population and most studies have been conducted in specific [2–6] or mixed populations [7–9], such as elderly subjects [2], pregnant women [3], and subjects with type 2 diabetes [4], asthma [5], or depressive disorder [6]. Many questionnaires used to assess the prevalence of GERD are not reproducible or have not been previously validated [2–4, 9–17]. Also, the survey approach, including telephone interviews, postal questionnaires, and self-completed instruments, has methodological limitations [3, 11, 12, 14, 16–25].

Therefore, to overcome these limitations, we conducted a population-based study to assess the prevalence of GERD in the general adult population of Temuco, a city in southern Chile, using a validated and reliable questionnaire specific for reflux disease [26]. Data of previous prevalence studies of GERD using face-to-face interviews were compared with findings of our study. The objective of our study was to determine GERD prevalence in a general adult population from Temuco in southern Chile and its association with demographic variables.

## Materials and methods

### Data source

The source population consisted of all individuals 18 years or older living in the urban area of Temuco, Chile, between 1 March 2017 and 31 December 2017. According to Chile’s Institute of Statistics (INE), the total urban population of the city was 232,528 inhabitants, of whom 172,116 subjects were adults (>18 years of age) [27].

### Study design and participants

A cross-sectional survey was designed, the primary objective of which was to determine the prevalence of GERD in the adult urban population of Temuco. Secondary objectives were: (i) to describe clinical features and associated factors of patients with GERD and (ii) to assess demographic, anthropometric, and sociocultural factors of respondents to the survey. Subjects of both sexes, aged 18 years or older who gave written informed consent, were included in the study. Exclusion criteria were as follows: morbid obesity, scleroderma, pulmonary disease, presence of neoplasms, esophageal motor disorders, diseases capable of causing GERD or alterations in esophageal motility as part of its natural history, benign and malignant gastroduodenal diseases, history of surgery of the upper gastrointestinal tract, and caustic injury. Pregnant women and subjects with cognitive impairment or mental illness were also excluded.

### Study procedures

A validated and reliable specific questionnaire for the diagnosis of GERD was used [26, 28]. The questionnaire consists of seven items: heartburn, regurgitation, chest pain, dry cough, dysphagia, dysphonia, and asthma. Heartburn, regurgitation, chest pain, and dry cough are scored from 0 to 3 (0: none; 1: at least once a month; 2: at least once a week; 3: every day) and dysphagia, dysphonia, and asthma from 0 (absent) to 1 (present), with a total score from 0 to 15. The questionnaire has been previously validated against the results of 24-hour esophageal pH monitoring and showed an internal consistency of 0.75 and inter-observer reliability of 0.87 [28]. Using a cut-off score of 3, the sensitivity, specific, positive, and negative predictive values were 92%, 95%, 98%, and 79%, respectively, with a correct classification of subjects of 92.4% [26, 28]. Other data of interest were added to the questionnaire, including age and gender, anthropometric data (height, weight, and body mass index [BMI]), tobacco and alcohol consumption, medications used for the control of GERD symptoms, and use of healthcare resources for GERD-related consultations.

### Sampling and administration of the questionnaire

**Figure 1.**
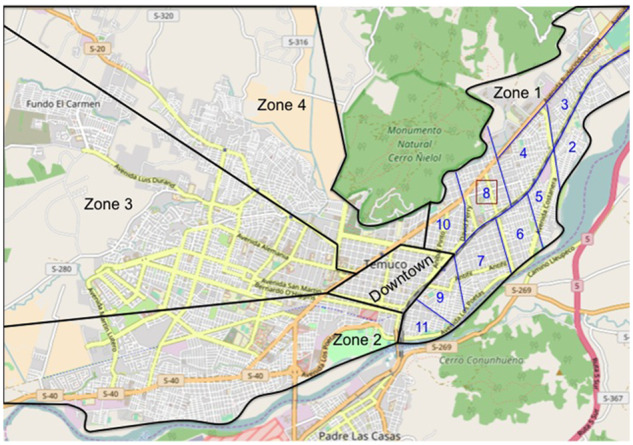
Map of Temuco used for further cluster randomization. Temuco was divided into four sectors properly representing the various economic and cultural areas of the city. Cluster randomization of sector 1 can be seen. The boxes in bold represent the subsectors studied.

**Figure 2.**
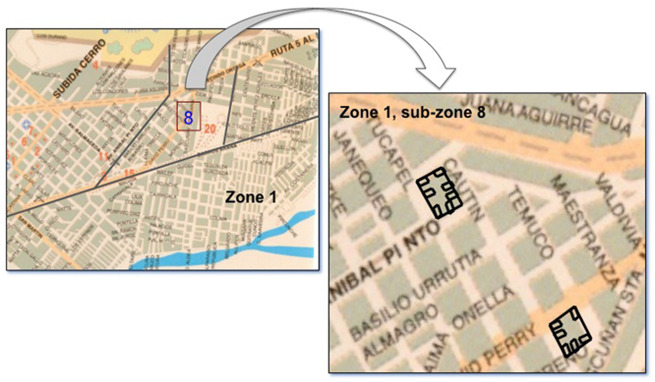
Sampling of subsector 8 of sector 1. Maps were raised to later randomize blocks and households.

The urban area of Temuco was divided into four sectors, which were representative of different economic and cultural areas of the city, e.g. sectors 1 and 2 are of medium social and economic status, sector 3 of high status, and sector 4 of low status (Figure 1). The downtown district was excluded because most of the buildings were commercial properties or office spaces. Sectors were then divided into 12 subsectors of approximately clusters of 10 blocks each. A two-stage cluster random-sampling method was used, in which a subset of households within each selected conglomerate of blocks was randomly selected for inclusion in the study (Figure 2). A team of six previously trained medical students performed face-to-face interviews in the selected households. They also confirmed the eligibility criteria, obtained the written informed consent, and administered the questionnaire. Interviewers were totally familiar with the instrument and knew how to explain better any eventual question that has not been fully understood by the subjects interviewed. When no one fulfilled the inclusion criteria, the household on the right of the index home was selected. When no one lived in the household, the interviewer returned 24 hours later and, if it was still uninhabited, it was replaced by the contiguous household on the right. Respondents who, according to the scale, were found to suffer from GERD were referred to the healthcare center for further consultation.

### Ethical consideration

The study was approved by the Ethics Committee of the Universidad de La Frontera. Written informed consent was obtained from all participants. Personal data were anonymized. This manuscript was written following the STROBE statement.

### Sample size and statistical analysis

Considering that the urban adult population of Temuco is 172,116 inhabitants [27] and based on a prevalence of GERD of 52.8% found in a previous pilot study of our group [29], with an accuracy of 3.0%, a confidence interval of 95%, and a design effect of 1.15, a sample size of 1,057 subjects was calculated. Categorical variables are expressed as frequencies and percentages, and continuous variables as mean and standard deviation or as median and range. The prevalence of GERD and the prevalence of individual symptoms of reflux disease were calculated. The chi-square test and the Fisher’s exact test were used for the comparison of categorical variables, and the Mann–Whitney *U* test or the Wilcoxon test for the comparison of continuous variables. Multiple logistic-regression analysis was used to assess variable independently associated with GERD. Statistical significance was set at *P *<* *0.05.

## Results

A total of 1,069 subjects were interviewed (47.9% women) with a median age of 40 years (range 18–92 years). The average BMI was 25.7 ± 4.4 kg/m^2^ (range 15.8–54.1 kg/m^2^). Education level included primary education in 18.1% of subjects, secondary education in 47.0%, and university studies in 34.3%. Six people (0.6%) were illiterate. Current smoking was recorded in 5.5% of subjects and alcohol consumption in 3.0%. Working status included active workers in 45.5% of subjects, housewives in 17.2%, pensioners/retired in 6.0%, unemployed in 3.0%, and students in 28.2%.

The median overall score of the in-study population was 2 (range 0–13) and the average score was 2.7 ± 2.6. On the other hand, 590 subjects (55.2%) had a score of <3 (average 0.9 ± 0.8) and 479 subjects (44.8%) had a score of ≥3 (average 5.1 ± 2.1). So, the prevalence of GERD was 44.8%. The prevalence of GERD varied according to areas surveyed (Table 1), but differences were not statistically significant. On the other hand, regurgitation was the most frequently reported symptom (54.8%) followed by heartburn (31.3%), cough (30.4%), and chest pain (29.8%) (Table 2).

**Table 1.** Distribution of the prevalence of gastroesophageal reflux disease (GERD) in the population surveyed from the four sectors of the city

Sector	No. of subjects	Presence of GERD, *n* (%)
1	361	142 (39.3)
2	232	128 (55.2)
3	231	114 (49.3)
4	245	95 (38.8)
Total	1,069	479 (44.8)

**Table 2.** Distribution of symptoms of gastroesophageal reflux disease (GERD) in the population surveyed (*n *=* *1,069)

Symptom	No. of subjects (%)
Heartburn	335 (31.3)
Regurgitation	586 (54.8)
Chest pain	258 (24.1)
Dry cough	318 (29.8)
Dysphagia	325 (30.4)
Dysphonia	241 (22.5)
Asthma	159 (14.9)

About one-third of respondents (*n *=* *353) with GERD scoring ≥3 in the questionnaire reported that they were using medications to control GERD symptoms, including antacids in 41.9% of cases, H_2_ antagonists in 30.9%, and PPIs in 26.9% (no relationship between positivity questionnaire and PPIs use was verified). Drugs had been prescribed by a healthcare professional in only 53.0% of subjects (*n *=* *187). Also, 68.0% of these subjects (*n *=* *240) never visited the public healthcare system (55.0% were visited in private practices, 27.5% self-medicated, 14.2% chose alternative medicines, and the remaining 3.3% did not respond to this item).

In the bivariate analysis, the prevalence of GERD was higher in women than in men (64.3% vs 26.9%, *P *<* *0.001). Also, subjects with GERD were older and shorter (Table 3). In the multivariate analysis, age (*P *<* *0.001) and female gender (*P *=* *0.001) were the variables independently associated with GERD.

**Table 3.** Association between demographic variables and gastroesophageal reflux disease (GERD)

Variable	No GERD (*n* = 590)	GERD (*n* = 479)	*P*-value
Age, years	40.7 ± 18.0	45.7 ± 18.0	<0.001
Sex, *n* (%)			<0.001
Female	183 (35.7)	329 (64.3)	
Male	407 (63.1)	150 (26.9)	
Height, cm	164.1 ± 13.6	161.6 ± 12.5	0.047
Weight, kg	68.7 ± 12.5	68.6 ± 12.7	0.942
Body mass index, kg/m^2^	25.6 ± 3.9	26.8 ± 4.9	0.162

## Discussion

The present population-based study carried out in the city of Temuco, Chile has shown a prevalence of GERD of 44.8%. We used a reliable and validated questionnaire for assessing esophageal reflux disease according to the presence and frequency of seven common symptoms of GERD. The prevalence found in the present study is lower than that observed in a previous cross-sectional survey (52.8%) in 2002–2003 in the same city, although, in this case, only 364 persons were interviewed [29]. A review of prevalence studies of GERD in adult populations using face-to-face interviews and published from 2000 is summarized in Table 4 [15, 29, 30, 32–36, 38–40]. Prevalence rates are highly variable from 3.5% to 52.8% as well as the number of subjects included in the surveys (from 364 to 13,959) and the geographical origin of the study samples. In these studies, the GerdQ questionnaire developed by Jones *et al*. [41] and the GERD questionnaire developed by Locke *et al*. [31] were the tools most frequently used for diagnosing GERD. In some surveys, however, the prevalence of GERD was defined as the presence of acid regurgitation and/or heartburn at least once a week [33, 37]. Marked differences in the prevalence rates of GERD have been reported in populations from Asia (between 1.7% and 27.5%) [25, 32–37, 40], Europe (between 14.8% and 28.7%) [16, 20, 42], and Latin America (between 4.7% and 52.8%) [22, 29, 30].

**Table 4.** Summary of GERD-prevalence studies in adult population using face-to-face interviews since 2000

Reference	Year	No. of subjects	Country	Population	Sampling method	Instrument	Validity	Reliability	Heartburn prevalence^a^ (%)	Regurgitation prevalence^a^ (%)	GERD prevalence (%)
Dacoll [30]	2000	1,141	Uruguay	Urban population	Systematic sampling	GerdQ [31]	Yes	NR	10.7	8.1	4.7
Wang [32]	2004	2,789	China	Adults of Xi'an	Stratified sampling	Unknown questionnaire	No	No	4.1	7.8	17.4
Cho [33]	2005	1,902	Korea	Asan-si residents	Simple random	Unknown questionnaire	Yes	Yes	2.0	2.0	3.5
Manterola [29]	2005	364	Chile	Urban adult population of Temuco	Cluster sampling	Manterola [26]	Yes	Yes	46.4	59.6	52.8
Moraes-Filho [15]	2005	13,959	Brazil	Urban adult population of 22 cities	NR	Unknown questionnaire	No	No	11.9	NR	7.3
Mungan [34]	2012	8,143	Turkey	Turkish general population	Simple random	GerdQ [31]	Yes	Yes	NR	NR	27.6
Shaha [35]	2012	2,000	Bangladesh	Urban and rural areas of northeastern Bangladesh	Simple random	Manterola [26]	Yes	Yes	5.25	5.25	5.5
Yönem [36]	2013	1,345	Turkey	Adults living in Sivas	Simple random	Locke [37]	Yes	NR	8.0	8.9	19.3
Bor [38]	2016	1,065	Russia	Adults in Moscow	Simple random	Locke [37]	Yes	NR	17.5	17.6	23.6
Wang [30]	2016	1,072	India	People in southern India	Multi-stage cluster	GerdQ [31]	Yes	NR	26.4	18.1	22.2
Bor [39]	2017	3,214	Turkey	Subjects aged 20 years or older from 17 cities	Three-stage stratified cluster	Locke [37]	Yes	Yes	12.7	18.7	22.8
Manterola	2020	1,069	Chile	Urban adult population of Temuco	Two-stage cluster random sampling	Manterola [26]	Yes	Yes	31.3	54.8	44.8

a Symptom present at least once a week; GERD: gastroesophageal reflux disease; NR: not reported.

The comparison across studies is limited, for different reasons: the heterogeneity of the in-study populations and the use of different scales and instruments with the same purpose, but only some of them valid and reliable [26, 31, 41]. On the other hand, there are a lot of studies using different definitions of GERD and utilizing ‘unknown questionnaires’ as measurement instruments. The majority of published instruments (almost 40) did not fulfill all relevant diagnostic criteria [43]. Finally, other reasons that may be associated are the form of applying the measurement instruments (face to face, by phone, mail, etc.), the accuracy of the sample-size estimation, the type of sampling strategies utilized, etc.

A high prevalence of main GERD symptoms was found in our study, particularly heartburn and regurgitation, although this finding is consistent with the high number of subjects who reported taking anti-reflux medications for the control of symptoms, generally prescribed by the public healthcare system. Also, age, female gender, and height were associated with GERD. A relationship of GERD with increasing age has also been reported in other studies [20, 33, 40]. It has been shown that non-erosive reflux disease is more common in women, whereas Barrett’s esophagus and esophageal adenocarcinoma are more frequent in men [44]. The estrogen-related endocrine milieu is reported to modulate the metabolism of fat and obesity is a main risk for GERD [45].

Obesity and BMI are well-known risk factors for reflux disease (especially in the Western population). This association is not coincidental because GERD pathophysiology is, in part, connected with overweight and obesity [17, 38, 45–47]. A relationship between BMI and GERD was not observed probably because one of the exclusion criteria of our study was obesity, in spite of which, the study population was slightly overweight (the BMI average was 25.7 ± 4.4 kg/m^2^, with a fairly similar distribution across the four sectors). The average of <3 and ≥3 points subgroups were 25.6 ± 3.9 and 26.8 ± 4.9 kg/m^2^, respectively (*P = *0.1619). Related to any dietary elements in this geographical area of Chile that could lead to obesity, we can argue that effectively there are some, e.g. the fruit of the araucarias (La Araucanía Region typical tree), called ‘piñones’; however, there is not enough evidence to asseverate it.

We did not find differences in other factors that have been related to the disease, such as smoking or alcohol consumption [48, 49]. The small percentage of smokers and alcohol users in our study may account for this finding. On the other hand, the proportion of subjects with university studies was 34.3%—a high rate that can be explained by the presence of numerous education centers in two areas of the city. The percentage of unemployed patients was also high because students accounted for 28.0% of the study sample. An influence of the education level or working status on the development of GERD was not established. After logistic-regression analysis, only age and female gender were significantly associated with GERD.

The potential effect of indigenous elements in the population samples is difficult to determine precisely because, although the self-declared indigenous population in urban Temuco in the 2017 census was 24.7% (including different races of original peoples) [50], there is no form to determine the proportion of indigenous elements in the different in-study sectors (possibly this value can be even higher, because there are people with indigenous ancestry who are not considered as such). On the other hand, regarding the potential influence of migrants from rural communities around introducing higher *Helicobacter pylori* rates and hence less GERD, there is evidence supporting no relationship between GERD and *H. pylori* presence; also, successful eradication therapy does not have an impact on the emergence or exacerbation of GERD [51–54].

Another interesting point to comment on is the potential association between the high rates of GERD and esophageal cancer (Barrett's-related) rates in the community. First of all, we do not know the reasons to explain the high prevalence of GERD found, but it is similar to a previous study published in 2005 [29]. On the other hand, the crude mortality rate of esophageal cancer per 100,000 inhabitants in Chile in 2015 was 3.7. However, in La Araucanía Region (Capital Temuco), it was 9.5 for men and 6.2 for women (the highest in the country) [55], so is a fascinating issue for further investigation. However, Barrett esophagus in our city has unknown incidence.

The present findings should be interpreted taking into account the limitations of the study, particularly the generalizability of the results to different target populations. The strengths of the study include the use of a validated and reliable questionnaire for assessment of GERD symptoms, trained interviewers, the face-to-face approach for administering the questionnaire, and the cluster random sampling for the selection of participants.

In conclusion, using a population-based cross-sectional study and a validated questionnaire, the prevalence of GERD in an urban area of southern Chile was 44.8%. An independent association of GERD with female gender and age was observed. Further studies using endoscopic examination would be necessary to ascertain the association between a positive questionnaire score for GERD and endoscopic esophagitis. In addition, it would be advisable to carry out education campaigns to detect and treat patients with GERD in order to prevent complications and to contribute to reducing the economic burden of the disease.

## Authors’ contributions

C.M. and L.G. made substantial contributions to the conception, design, analysis, and interpretation of the data, and drafted the manuscript. T.O. and G.D. made substantial contributions to the design, analysis, and interpretation of the data. All authors read and approved the submitted version.

## Funding

This work was partially supported by Project MEC 80170022, CONICYT Chile 2018. The funding body collaborated in the design of the study and interpretation of data and in writing the manuscript.
